# Anti-n-Methyl-d-Aspartate-Receptor (NMDAR) Encephalitis in Association with Ovarian Teratoma

**DOI:** 10.7759/cureus.1425

**Published:** 2017-07-05

**Authors:** Javaad Ahmad, Muhammad Saad Sohail, Amina Khan, Ahmed H Qavi, Pramod Gaudel, Mehr Zahid, Salman Assad

**Affiliations:** 1 Department of Neurology, Mount Sinai Hospital New York, Usa; 2 Shifa International Hospital, Shifa International Hospital, Islamabad, Pakistan; 3 Shifa Tameer E Millat University, Shifa International Hospital, Islamabad, Pakistan; 4 Department of Medicine, Mahroof Hospital; 5 Department of Medicine, Montefiore New Rochelle Hospital, Albert Einstein College of Medicine, Ny, Usa; 6 Internal Medicine, University of Lahore, Lahore, Pakistan; 7 Department of Medicine, Shifa International Hospital, Islamabad, Pakistan

**Keywords:** ovarian teratoma, nmda, nmdar encephalitis, anti-nmdar, anti-nmda receptor encephalitis

## Abstract

Anti-N-methyl-D-aspartate-Receptor (NMDAR) encephalitis is an autoimmune disorder with a multifaceted presentation that involves memory deficits, psychiatric symptoms, and autonomic instability. This case report describes the classic presentation of Anti-NMDAR encephalitis and highlights its association with ovarian teratomas. We present a 26 -year-old female who came in with new onset seizures and altered mentation who subsequently developed automatism. Electroencephalograms (EEG) showed left frontal spikes and right temporal delta activity. Magnetic resonance imaging (MRI) revealed right temporal hyper-intensity. The diagnosis was established with positive anti-NMDAR antibodies in the cerebrospinal fluid (CSF). The patient was initially treated with steroids and valproic acid, however, her condition progressively worsened. A five-day course of intravenous immunoglobulins (IVIG) was started followed by rituximab. The clinical course was complicated with the patient developing neutropenic fever and cerebrospinal fluid cultures (CSF) growing methicillin-sensitive Staphylococcus aureus (MSSA). She underwent pelvic imaging which showed a right ovarian teratoma. Evidence suggests that removal of ovarian tumor leads to better clinical and mortality outcomes in patients with Anti-NMDAR encephalitis. It is important for the internist to consider paraneoplastic syndromes in patients with Anti-NMDAR encephalitis.

## Introduction

Anti-N-methyl-D-aspartate-receptor encephalitis was initially described by Dalmau and colleagues in 2007 when they discovered the anti-N-methyl-D-aspartate (NMDA) receptor antibody in a set of females who had an array of neurological symptoms (short-term memory loss, followed by psychiatric symptoms or confusion and a decreased level of consciousness) in association with ovarian teratomas [1]. The patients can present with a viral-like prodromic syndrome [1], which can be initially confused as viral encephalitis, much like our case. Recognizing the syndrome is crucial as most of the cases can achieve better clinical and mortality outcomes by the removal of the teratoma and immunotherapy [2]. In our report, we describe a similar presentation of a female patient with an ovarian teratoma that was successfully managed after being misdiagnosed as infectious encephalitis. Informed consent statement was obtained for this study.

## Case presentation

**Figure 1:**
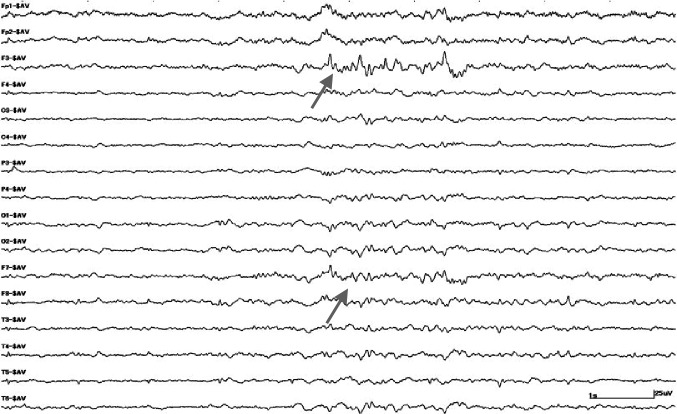
Electroencephalogram image showing spikes in the left frontal area Spikes in the left frontal area is indicated by the grey arrows

A 26-year-old female presented with new onset seizures and bizarre behavior. Review of systems was otherwise unremarkable. Her social history was significant for alcohol intake and surgical history included an abortion with intra-uterine device (IUD) placement three months back. On examination, the patient was hemodynamically stable. She was oriented to time and place but not to a person. Shortly thereafter, she developed automatisms with back and forth body movements. Initial electroencephalogram (EEG) showed left frontal spikes (Figure 1).

**Figure 2:**
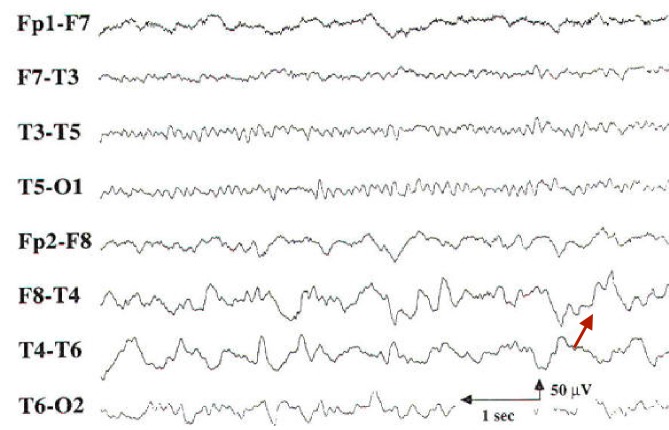
Repeat image of the electroencephalogram Persistent polymorphic delta activity in the right temporal region indicated by the red arrow

She continued to have seizures and disturbed behavior. A repeat EEG was performed which revealed right temporal delta activity (Figure 2).

EEG was discontinued due to a seizure-free interval of 24 hours. Magnetic resonance imaging (MRI) of the brain was performed, which showed right temporal hyper-intensity. She was treated with acyclovir prophylaxis due to suspected herpes simplex virus (HSV) encephalitis. However, both HSV serology and HSV deoxyribonucleic acid (DNA) in the CSF by polymerase chain reaction (PCR) were negative. Further workup revealed serum and cerebrospinal fluid to be positive for Anti-NMDAR antibodies and increased leukocytes with neutrophilic predominance, because of which, prophylactic vancomycin and steroids were started. Due to the progressive decline in mental status and unresponsiveness, steroids were discontinued. She was also started on valproic acid (VA), which had to be increased to 750 mg 12 hourly due to progressive psychosis. The dose of VA had to be increased again to 1000 mg 12 hourly [as latest VA levels were 53 µg/mL (therapeutic 50-125 µg/mL)] to better manage worsening agitation and psychosis. In addition, she was given a five-day course of intravenous immunoglobulins (IVIG) with a further plan to start rituximab. However, after the first dose of rituximab, she developed neutropenia, fever, and tachycardia. CSF NMDA titers were 1:840. Prophylactic vancomycin was discontinued after CSF cultures were positive for methicillin-sensitive Staphylococcus aureus (MSSA), for which methicillin was started. Over the course of 24 hours, she developed opisthotonic posturing, fever, agitation, and tachycardia. Glycopyrrolate was started for increased secretions.

Due to the association of anti-NMDAR antibodies with ovarian teratomas, a transvaginal ultrasound was performed which was suggestive of a 2 x 3 cm right ovarian cyst. Magnetic resonance imaging (MRI) of the pelvis further confirmed the presence of non-malignant transformation. Eventually, due to the deteriorating condition of the patient, right oophorectomy was planned. Her condition remained the same even after the unilateral oophorectomy, which stabilized after the second oophorectomy was done.

## Discussion

In 1997, a report of a young female described anti-NMDA-receptor encephalitis initially. This young female presented with an ovarian teratoma and symptoms that included psychiatric manifestations. There was a gradual significant improvement in symptoms after tumor removal [3], much like our case. In 2005, a series of four females with an ovarian teratoma, psychiatric symptoms, altered level of consciousness, and central hypoventilation was described. Authors made a hypothesis that the symptoms were due to a paraneoplastic process which most likely occurred due to an antibody to an unknown antigen expressed in the hippocampus [4-5]. The associated antibody were later discovered to be anti-N-methyl-D-aspartate (NMDA) receptor antibody in 2007 [1]. To date, a large case series characterized more than 500 cases of anti-NMDAR encephalitis, which also stated that 81% of the patients affected were females, with a median age of diagnosis of around 21 years of age [6].

Antibodies to multiple synaptic targets have been identified in the patients with symptoms of encephalitis. These include the glutamate receptors GluA1 and GluA2, subunits of the alpha-amino-3-hydroxy-5-Methyl-4-isoxazolepropionic acid receptor (AMPAR) and the B1 subunit of the aminobutyric acid-B receptor (GABABR). However, the most common form of autoimmune encephalitis with the loss of self-tolerance to synaptic proteins occurs with detectable autoantibodies against the N-methyl-D-aspartate receptor (NMDAR). Autoantibodies directed against the NR1 subunit of the NMDAR are thought to be responsible for this particular pathology [7]. It has been shown that these autoantibodies result in autophagy of NMDARs causing a decrease in number. This form of the disease was officially identified as “anti- NMDAR encephalitis” by Dalmau and colleagues in 2007 [1].

This syndrome usually begins with viral-like symptoms including a headache, nausea, vomiting, fever, and fatigue. Since these symptoms are particularly non-specific, it’s usually not recognized as a prodrome unless the illness progresses with a spectrum of neuropsychiatric symptoms. These symptoms have been divided into early and late stage symptoms. Early stage symptoms generally present with two weeks of prodromal symptoms and include confusion, hallucinations, memory loss, mood disturbances, anxiety, self-harming behaviors, paranoia, seizures and movement disorders such as facial twitching and choreoathetosis [5]. Since psychiatric symptoms are most often prominent, 77% of patients are initially seen by psychiatrists [5]. These patients don’t respond to conventional antipsychotics and may progress to late stage symptoms which include hypoventilation, autonomic instability, and decreased responsiveness. The patients might even have hyper or hypotension, urinary incontinence, and hyperthermia [5].

The median age of onset of symptoms is 21 years, although cases have been reported in patients ranging from eight months to 85 years of age [5-6]. Teratomas are found in large numbers of patients, most commonly in women between ages 12 and 45 years and in the patients of Asian or African American descent [5-6,8]. Most commonly, these are ovarian teratomas, although other germ-cell and rarely non-germ cell tumors have also been described in association with anti-NMDA-receptor encephalitis [6].

Cerebrospinal fluid (CSF) analysis is required for the diagnosis of this disorder. CSF shows elevated protein and moderate lymphocytic pleocytosis. In addition, almost all the patients have antibodies in the serum or CSF that recognize the NMDA receptor, which confirms the diagnosis [8]. After the diagnosis, which can be tricky, the condition is treated with a focus on immunotherapy and removal of the tumor if it is present. The best thing to do is to remove the tumor and treat the patient with corticosteroids and intravenous immunoglobulin (IVIG). Plasma exchange can also be done for treating the immune response. IVIG is preferred in the patients who are agitated as plasma exchange is difficult in these set of patients [2]. The mortality rate is around 4%. However, around 75% of the patients recover fully or have mild neurological deficits, whereas 25% may face severe disabilities and die. The patients who do not have an underlying tumor also require treatment with rituximab or cyclophosphamide along with the first line immunotherapy. In addition, continued immunosuppression with azathioprine or mycophenolate mofetil is suggested for one year along with periodic screening for an ovarian teratoma for two years in these patients [2].

## Conclusions

Anti-N-methyl-D-aspartate (NMDA)-receptor encephalitis is a rare syndrome which presents with neurological symptoms like seizures and can be confused with infectious encephalitis. It can present as a paraneoplastic syndrome with ovarian teratomas or other ovarian tumors. Favorable outcomes may be achieved with the removal of the tumor and adjunctive immunotherapy. The patients may also require extensive monitoring for development of teratoma, and should also be counseled on appropriate contraceptive use if remaining on long-term immunosuppression.
